# Encephaloid Cancer of the Liver

**Published:** 1870-11

**Authors:** T. G. Williams

**Affiliations:** Watertown, Wis.


### ENCEPHALOID CANCER OF THE LIVER.

WATERTOWN, WIS., *Oct. 8, 1870.*

DEAR "EXAMINER,"—If you think it of sufficient interest you may record the following:

Mar. 28th.—J. R., age 50, farmer, called at my office for treatment. Said he had been troubled at times, for years, with distress and sensation of burning in epigastrium. Had taken various remedies from different ones for "biliousness" and "worms," sometimes with apparent relief, more frequently with none. He had for some time past been troubled much more than usual.

He then complained of constant burning in stomach, and was not able to eat certain kinds of food, owing to increased distress produced. Tongue constantly coated and obstinate constipation. General health merely normal; working some, and walked on that day 15 miles. Could not sleep on back or left side. Never troubled with vomiting, though lately he would regurgitate certain kinds of food. Careful examination was made, owing to peculiar history. Nothing found with stomach different from what we usually find in chronic folliculitis of that organ. Liver seemed a little enlarged, still not so much as to be certain of it.

Not satisfied with what I found, I told him to return in two weeks, and gave him directions for improving digestion, etc.

April 12th.—Came again. He was quite satisfied with result of treatment. No change in size of liver. Less tenderness over stomach. Constipation disappearing; appetite better, but still not able to sleep on back or left side. Made few changes in treatment, and told him to call again if not better.

May 4th.—Was called to see him. Found him anxious about extreme anasarca of lower half of body, and almost complete anuria, which had taken place the preceding night. Said that he had continued feeling much better since his call at my office, and thought he "would be all right if he could get rid of the *dropsy.*" That night he felt that he could sleep on his back, and thought he would try it. While attempting to dress he found his feet and legs greatly enlarged. As the œdema was confined to lower half of body, I searched for a local obstruction to venous circulation of vena cava. This I found in enlarged liver, the border of which could be distinctly felt below the ribs; hepatic dulness, extending over a much larger surface than at first. No pain; countenance anxious; skin yellow; eyes fearfully white. Loss of appetite and some nausea. Ordered a hot-air bath, a mild diuretic, and that he should be constantly on his right side, regarding the symptoms as due to pressure of the enlarged liver on the vena cava. Seeing a hopeless case of cancer I could do nothing but palliate symptoms.

May 6th.—Found patient and friends hopeful, as anasarca was greatly diminished, and urine free and normal. The liver, however, had extended over one-half inch lower in two days, and nodules could be felt in several places over it. Told the friends what termination would be.

May 8th.—Some favorable changes in patient's symptoms, but still an increased decent of one inch. Hoping that my diagnosis was exaggerated, they wished for counsel.

May 10th.—Brought Dr. Cody, who confirmed what had been said. Lower border of liver not far from umbilicus; cachexia decided. Not much anodyne needed throughout the treatment. This rapid growth continued till the abdominal cavity was nearly filled.

Hiccough set in about the 14th, but was controlled with arom. spits. am. to a great extent. Would take no medicine towards the last but this, for the distress from the hiccough was very irritable, and became quite helpless from œdema till the 22nd, when he died, 18 days from the time the liver began rapidly to enlarge. No autopsy; abdomen at death was greatly distended, hard, and nodular.

The interesting features of this case are: obscure approach, with apparent amelioration for 5 weeks, under hygienic and dietitic regulation, rapid growth, immense size, and freedom from great pain. T. G. WILLIAMS.

